# Investigating the Use of Impedance Flow Cytometry for Classifying the Viability State of *E. coli*

**DOI:** 10.3390/s20216339

**Published:** 2020-11-06

**Authors:** Christian Vinther Bertelsen, Julio César Franco, Gustav Erik Skands, Maria Dimaki, Winnie Edith Svendsen

**Affiliations:** 1 DTU Bioengineering, Technical University of Denmark, Søltofts Plads 221, 2800 Kgs. Lyngby, Denmark; jucesarfranco@gmail.com (J.C.F.); madi@dtu.dk (M.D.); wisv@dtu.dk (W.E.S.); 2 SBT Instruments A/S, Symfonivej 37, 2730 Herlev, Denmark; ges@sbtinstruments.com

**Keywords:** impedance spectroscopy, impedance flow cytometry, bacteria detection, bacteria characterization, bacteria inactivation, lab-on-a-chip

## Abstract

Bacteria detection, counting and analysis is of great importance in several fields. When viability plays a major role in decision making, the counting of colony-forming units grown on agar plates remains the gold standard. However, because plate counts depend on the growth of the bacteria, it is a slow procedure and only works with culturable species. Impedance flow cytometry (IFC) is a promising technology for particle detection, counting and characterization. It relies on the perturbation of an electric field by particles flowing through a microfluidic channel. The perturbation is directly related to the electrical properties of the particles, and therefore provides information about their composition and structure. In this work we investigate whether IFC can be used to differentiate viable cells from inactivated cells. Our findings demonstrate that the specific viability state of the bacteria has to be considered, but that with proper characterization thresholds, IFC can be used to classify bacterial viability states. By using three different inactivation methods—ethanol, heat and autoclavation—we have been able to show that the impedance response of Escherichia coli depends on its viability state, but that the specific response depends on the inactivation method. With these findings we expect to be able to optimize IFC for more reliable bacteria detection and counting in the future.

## 1. Introduction

There is an increasing need for fast and accurate quantification of bacteria concentrations in multiple fields such as environmental monitoring and hazard analysis in multiple industries, particularly the food industry. The purpose of the quantification is to ensure that the total bacteria concentrations in the production adhere to the regulations and guidelines set by national and international authorities and to ensure safe and high-quality products. Monitoring happens by collecting samples of cleaning water (e.g., CIP-water (clean-in-place) from production equipment) or by collecting samples from production surfaces [1] (e.g., using a moist swab) and resuspending the collected bacteria in an appropriate buffer liquid. The bacterial concentrations in the collected samples are then analyzed using plate counting [2]. This method is well established and reliable, but is notoriously slow and only shows the presence of culturable bacteria [3]. Consequently, a need exists for a fast and reliable method for the quantification of bacteria concentrations in samples that can complement or replace traditional plate counting. 

In recent years, a number of alternative technologies for the quantification of bacteria concentrations has emerged to overcome the limitations of traditional plate counting. Some examples are polymerase chain reaction-based (such as real-time PCR or qPCR) or fluorescence-based techniques (such as fluorescent-assisted cell sorting). However these methods are labor-intensive and require complicated sample processing with expensive reagents [4]. Impedance Flow Cytometry (IFC) is an emerging technology for single-cell analysis and particle enumeration [5]. This technology has shown its potential for single-cell analysis in samples containing a wide range of cell types. It has been used to characterize the electrical properties of Red Blood Cells (RBCs) [6], to analyze plant spores [7], to identify the differentiation state of Mesenchymal Stem Cells (MSCs) [8], to characterize and classify tumor cells [9], to study the effect of chemotherapy treatment on the cell membrane of HeLa cells [10], to monitor the viability of yeast cells [11] and to analyze pollen samples [12]. Due to their small size, it can be difficult to achieve sufficient signal-to-noise when measuring bacteria using IFC. Several suggestions have been given on how to improve this [13,14,15]. The spectral response of *E. coli* has previously been studied using impedance spectroscopy techniques [16,17], and while some research has gone into specifically investigating the use of IFC to detect bacteria [18,19], assess the viability of Bacillus megaterium [20] and to classify bacteria based on their species [21], a general characterization of bacteria using IFC remains an underdeveloped research area. 

In this paper we take a more general approach to the analysis of bacteria viability with IFC by including general inactivation methods such as ethanol and autoclaving. To our knowledge this is also the first time IFC has been used to study the viability of *E. coli*.

IFC has a number of advantages over competing methods for bacteria analysis, mainly that it is label-free, non-invasive, sensitive and fast. A traditional plate count will underestimate the true number of viable bacteria in non-laboratory cultures and environmental samples since it is not able to detect the presence of unculturable species or bacteria that are intact and metabolically active but unable to divide under ordinary conditions (viable but non-culturable bacteria) [22,23,24,25]. In IFC, every particle or cell in the samples is counted and characterized no matter what the species is or what viability state the bacteria is in. Consequently, IFC has the potential to provide a more accurate measurement of the total bacteria count by including cells which would previously have been unseen.

However, using IFC as a replacement for traditional plate counting may also introduce new technical challenges. When a bacterium is inactivated it loses its ability to divide and subsequently will not generate a visible colony (colony-forming unit, CFU) on an agar plate. In this case, the cell could retain enough of its cellular structure to still be counted using IFC and lead to a false positive detection of inactivated cells. It is therefore imperative that the change in dielectric response of inactivated bacteria is investigated and characterized, if IFC is going to emerge as a useful and reliable alternative to plate counting.

Despite being frequently used, the term bacteria viability does not have a straightforward definition [26]. A comprehensive definition of viability considers cell component integrity, metabolic activity and ability to proliferate. However, in this article we will use viability (i.e., “a viable cell”) to mean either a cell that shows an intact cellular structure (specifically the cell membrane) or is able to give progeny on an appropriate agar and generate visible colonies. Similarly, we will use the term “inactivated cell” to mean a cell that has undergone an inactivation process so that it no longer fulfills the viability conditions described above. The inactivation agents used in this study are ethanol, heat (at 90 °C) and autoclaving. These methods are expected to act with rapid and nearly simultaneous oxidation or denaturation of multiple targets, including the cytoplasmic membrane, proteins, ribosomes, and/or DNA [26].

In this paper we use a prototype multi-frequency impedance flow cytometer developed by the Danish company SBT Instruments A/S (Herlev, Denmark) to test the impedance response of *Escherichia coli* (*E. coli*). We inactivate the *E. coli* cells using three different methods (ethanol, 90 °C heat or autoclaving) and compare it to the impedance response of untreated *E. coli* cells. We also discuss the expected impact of the inactivation on the electrical properties of the bacteria. Furthermore, we compare the experimental impedance measurements with CFU counts and fluorescence imaging to determine whether the change in impedance response can be correlated to the cells’ ability to grow and the membrane integrity of the cells, respectively. Finally, we evaluate the sensitivity and specificity of IFC as a tool for characterizing inactivated and viable cells.

## 2. Materials and Methods

### 2.1. Impedance Flow Cytometry

**Figure 1:**
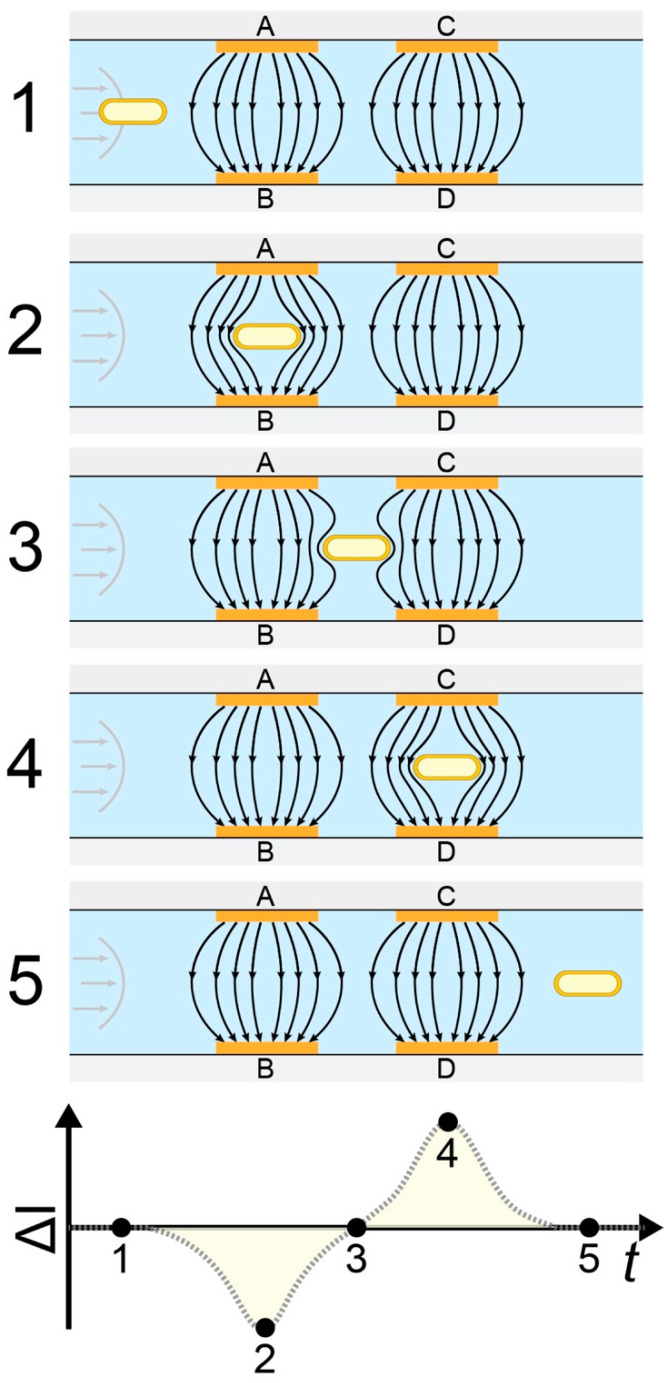
Detection principle. Schematic of 5 positions of a transitioning bacteria (1, 2, 3, 4, and 5) and the corresponding differential current (ΔI = I_AB_ − I_CD_). When a bacterium enters the detection area (position 1), the current between the two electrode sets is identical (I_AB_ = I_CD_), resulting in a differential current of zero. When the bacterium moves between electrodes A and B (position 2), the electric field is perturbed resulting in a non-zero differential current. When the bacterium is exactly between the electrode sets (position 3) the differential current is again zero. As the bacterium transitions between electrodes C and D and further out of the detection area, the electric field is again perturbed giving rise to a differential current (position 4). At position 5, the differential current is again zero as the bacterium exits the detection area.

The working principle of impedance flow cytometry (which has also been described in detail elsewhere [27,28,29]) is illustrated in Figure 1. An electrolyte with suspended particles and/or microorganisms was injected through a microfluidic channel by a pump. A set of 4 microelectrodes were located on the top and bottom (2 on the top and 2 on the bottom) of the microfluidic channel facing each other. A multi-frequency alternating current (AC) voltage was applied to the two electrodes on the top of the channel, giving rise to a current perpendicular to the direction of the flow, which was measured on the two electrodes on the bottom of the channel. In our case, we used two frequencies—a low frequency of 366 kHz and a high frequency of 6.9 MHz. When a particle or a bacterium passes between the electrodes, it changes the dielectric properties of the space between the top and bottom electrodes and causes a change in current. The difference in current between the two electrodes on the bottom of the channel was measured giving rise to a characteristic double gaussian event as shown in Figure 1. Each event was detected and analyzed in order to characterize the properties of each passing particle or bacterium. 

A more detailed description of the impedance flow cytometer and the event detection used in this work can be found in the Section 1.1 of Supplementary Materials.

**Figure 2:**
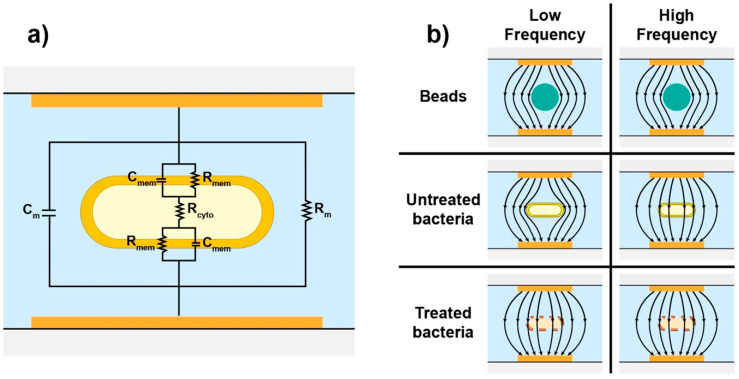
(**a**) Simplified equivalent circuit model of *E. coli* bacteria. The model is composed of components representing the electrolyte resistance and capacitance (R_m_, C_m_), the resistance and capacitance of the cell membrane (R_mem_, C_mem_) and the resistance of the cell interior (R_cyto_). (**b**) Electric field penetration in polystyrene beads and in bacteria with different viability states at low and high frequencies.

In order to understand the interaction between bacteria and the current flowing between the electrodes, it is common to represent the electrical properties of cells as electrical components in an equivalent circuit model (ECM) where the cell is modelled as a simple single-shelled particle [30] as seen in Figure 2a. An equivalent circuit is a simplified circuit which retains the electrical characteristics of the original physical system. The assumption in the ECM is that the complex permittivity and conductivity of a physical system can be replaced by equivalent electrical components [31]. 

A simple ECM of a bacteria suspended in a liquid between two electrodes has components representing the resistance and capacitance of the electrolyte (R_m_, C_m_), the resistance and capacitance of the cell membrane (R_mem_, C_mem_) and the resistance of the cell interior (i.e., cytoplasm) (R_cyto_) [32]. Electrolyte resistance and capacitance are material properties that depend on the ionic strength of the electrolyte, but also on the temperature of the solution. 

Figure 2b shows the expected electric field penetration in polystyrene beads as well as in treated and untreated bacteria. Polystyrene beads are often used as reference samples in IFC experiments and are expected to be electrically isolating at both low and high frequencies [21]. For a viable bacterium, electrical conductivity across the membrane is considered to be very low (i.e., high membrane resistance) [33]. Combined with a noticeably high membrane capacitance this means that the membrane offers a significant barrier to current flow even at relatively high frequencies in the range of 100–500 kHz. At higher frequencies, the membrane capacitance is effectively short-circuited allowing the electric field to probe the interior of the cell as illustrated in Figure 2b.

In this work we investigate how changes in membrane structure due to inactivation affects the electrical properties of the membrane and cell interior. During inactivation with either heat or ethanol it is expected that the membrane properties will change significantly, which in turn will affect both the effective membrane resistance and capacitance. Primarily, we expect that a disrupted membrane will have a significantly lower resistance allowing current to flow through the cell at lower frequencies as illustrated in Figure 2b.

### 2.2. Experimental Procedure

We grew Escherichia coli cells in tryptic soy broth and inactivated them using ethanol, heat and autoclavation in three separate experiments. In each experiment, we performed IFC measurements, plate counts and fluorescent microscopy on the inactivated sample as well as on an untreated control sample. A flow diagram of the sample preparation can be found in the Section 1.2 of the Supplementary Materials.

#### 2.2.1. Preparing Bacteria Samples

Escherichia coli (Migula) Castellani and Chalmers ATCC 8739 (Microbiologics^®^, Saint Paul, MN, USA) was inoculated on tryptic soy agar (TSA, Sigma Aldrich, St. Louis, MO, USA) plates using a resuspended lyophilized pellet following the manufacturer’s instructions. The plates were incubated overnight at 37 °C and stored in the fridge. 

For each experiment, a pre-culture was prepared by inoculating 10 mL of tryptic soy broth (TSB, Sigma Aldrich) with a single colony picked from the TSA plate. The pre-culture was incubated overnight in a shaking incubator at 37 °C and 200 RPM. The experimental culture was prepared by inoculating 40 mL of fresh TSB with 40 µL from the pre-culture (1:1000 dilution) and incubated again in a shaking incubator at 37 °C and 200 RPM until they reached the exponential growth phase (approximately 4 h for a final concentration of ~10^8^ CFU/mL). We used cells in the exponential phase to avoid a high degree of natural cell death which could occur in the later stages of growth.

#### 2.2.2. Bacteria Inactivation 

For the ethanol inactivation, 40 mL of experimental culture was split into two vials (2 × 20 mL) one for inactivation and one for control. The bacteria were harvested from each vial by centrifugation at 10,000 rpm for 5 min (Multifuge X3, Thermofisher). The sample for inactivation was resuspended in 20 mL of a 70% ethanol solution (70% *v*/*v*, TechniSolv, VWR chemicals). After 5 min of exposure to ethanol, the bacteria suspension was centrifuged at 10,000 rpm for 5 min and resuspended in fresh PBS (Dulbecco’s Phosphate Buffered Saline, Sigma-Aldrich). To ensure no ethanol was remaining in the solution, the bacteria suspension was centrifuged again at 10,000 RPM for 5 min and resuspended in PBS. The control sample was resuspended in PBS instead of ethanol but was otherwise taken though the same steps.

For the heat inactivation, bacteria were harvested from the 40 mL exponentially growing experimental culture by centrifugation at 10,000 rpm for 5 min and were subsequently resuspended in 40 mL fresh PBS. After that, 3 mL of resuspended bacteria were transferred to a glass vial. A block of aluminum with drilled holes for sample vials was placed on a hot plate at 90 °C. When the aluminum block reached 90 °C the sample vial was placed inside the block for 5 min and subsequently placed on ice for 5 min to cool down. The viable control sample was placed on ice for 5 min after being transferred to the glass vial and then kept at room temperature until further analysis.

For the autoclave inactivation, bacteria were harvested from the 40 mL exponentially growing experimental culture by centrifugation at 10,000 rpm for 5 min and subsequently resuspended in 40-mL fresh PBS. After this, 3 mL of resuspended bacteria were transferred to a 50 mL glass bottle. The bottle was placed in the autoclave and a standard cycle of 25 min was run (125 °C at 20 PSI). The control samples were kept in the fridge for the duration of the inactivation process.

#### 2.2.3. Impedance Protocol

Bacteria for the IFC measurements were harvested from the 40 mL exponentially growing experimental culture by centrifugation at 10,000 rpm for 5 min and subsequently resuspended in 40-mL fresh PBS. Subsequently, 30 µL of the bacteria sample were pipetted into a tube containing 3 mL of 1/20-diluted PBS (diluted with Milli-Q water), for a total reduction in concentration of 1/100.

The impedance measurements were carried out using a prototype impedance flow cytometer from SBT Instruments A/S (Herlev, Denmark). All measurements were performed using two simultaneous frequencies: 366 kHz and 6.9 MHz. Frequencies in this range have previously been shown to effectively characterize bacteria and non-bacteria [21]. The AC voltage applied to the electrodes was 15.8 V_pp_. The total measuring time per sample was 5 min. The current response was recorded with a sample rate of 23 kSa/s. An individual data stream was recorded simultaneously for the real and imaginary parts of the complex current for both the low and the high frequency. When the measurements were finished the data were transferred to a PC for further analysis.

A separate reference sample of polystyrene beads (1.5 µm, Polysciences, Inc., Warrington, USA) was analyzed during each inactivation experiment. The beads were prepared by diluting the stock solution of beads in 1/20-diluted PBS for a final concentration of ~1 × 10^6^ beads/mL. 

The concentration of cells (*C*) was calculated using the average transition time of the events (*t_event_*), the dimensions of the detection volume on the microfluidic chip (*w*—width of the channel, *h*—height of the channel, and *l*—distance from electrode edge to electrode edge), the total number of events during a measurement (*N*) and the total measurement time (*T*). The following equation was used to calculate the concentration:

(1) $$C=\frac{Nk\langle t_{event}\rangle }{whlT},$$

with *k* being a dimensionless constant calculated numerically that compensates for the parabolic flow profile in the microchannel.

A schematic overview of the experimental setup including the impedance flow cytometer can be found in the Section 1.1 of the Supplementary Materials together with a short description of the fabrication of the microfluidic chip and the event detection algorithms. 

#### 2.2.4. ROC Curves and Classification

In order to test the classification capabilities of the impedance flow cytometer we plotted receiver operating characteristic curves (ROC curves) showing the false positive rate (FPR, equal to 1-selectivity) and the true positive rate (TPR, equal to sensitivity) for varying thresholds. The sensitivity is defined as the ratio between the number of true positive events and the number of total positive events, and the selectivity is defined as the ratio between true negative events and the number of total negative events. In this work we define positive events as describing an intact cell. The sensitivity is therefore understood to be the ratio between the number of events characterized as viable bacteria in the untreated sample (true positives) and the total number of events in the untreated sample (total positives). Similarly, the selectivity is understood to be the ratio between the number of events characterized as not viable cells and the total number of events in the inactivated samples (or bead samples).

The curves were plotted using MATLAB. The threshold varied in steps from one extreme (100% FPR and 100% TPR) to the other (0% FPR and 0% TPR) and the corresponding FPR and TPR were calculated for each step. In our case, we chose the classification of a viable untreated bacterium as a positive classification. 

#### 2.2.5. CFU Counting

After the inactivation procedure, the inactivated samples were diluted 10 times by transferring 100 µL of the bacterial suspension into a microcentrifuge tube containing 900 µL of PBS. The control samples were further diluted by repeatedly vortexing and transferring 100 µL to the next tube until reaching a 10^−6^ dilution. The undiluted and last dilutions from each sample (both inactivated and control) were plated individually on separate TSA plates using an automatic pipette (Eppendorf Xplorer, Eppendorf). In total, five evenly spaced droplets of 20 µL from each sample were dispensed per plate [34]. After the drops on the agar dried, the plates were sealed with parafilm and incubated at 37 °C overnight. Colonies were manually counted in droplets with 6 to 60 colonies. The five drops for each dilution were considered to calculate the CFUs and the standard deviation for each initial sample.

#### 2.2.6. Fluorescent Imaging

Undiluted bacteria suspensions were stained after the inactivation process using the LIVE/DEAD^®^ BacLight™ Bacterial Viability Kit (Thermo Fischer Scientific, Waltham, MA, USA. The principle of the staining mix is based on the differential exclusion of the dyes according to membrane integrity. Bacteria with intact membranes show only green fluorescence while bacteria with compromised or disrupted membranes show green and red fluorescence.

A solution containing a 1:1 mixture of SYTO9 at 3.34 mM and propidium iodide at 20 mM was prepared and 3 µL were added to 1 mL of bacteria suspension. After 15 min of incubation in complete darkness, 5 µL of bacteria suspensions were deposited on top of a poly-L-Lysine-coated glass slide and covered with a 1 × 1 cm^2^ cover slide. The fluorescent samples were observed under the epifluorescence microscope (Heat and Ethanol: Axioplan 2, Zeiss; Autoclave: Olympus U-TV1X-2 + U-LH100HG) using two filters—FITC for the visualization of green fluorescence and a TRITC for the visualization of red fluorescence. Pictures were taken with the coupled camera (Heat and Ethanol: CoolSNAP-Procf Color, Media Cybernetics; Autoclave: XC30, Olympus) and image processing was performed using Fiji ImageJ [35] (see Section 1.3 of Supplementary Materials for further details). A different microscope was used to capture images of the autoclaved sample due to repeated problems with a low intensity in that particular sample. 

## 3. Results

Three different inactivation experiments were carried out using inactivation treatments with ethanol, heat and autoclavation, respectively. Shortly after each inactivation was completed, the untreated and treated samples were analyzed using the impedance flow cytometer and the concentration and electrical responses were obtained from the recorded data. The same samples were also analyzed using plate counting to investigate viability and fluorescent microscopy in order to investigate membrane integrity. 

### 3.1. Concentrations of Bacteria Samples

Bacteria concentrations were studied after the inactivation treatment using drop plating and IFC in parallel. The concentrations measured with drop plating and IFC for the untreated and treated samples from the three inactivation experiments can be found in Table 1. The concentration of bacteria in the untreated samples was roughly 10^8^ CFU/mL in all three experiments. No standardization of the concentration (e.g., using optical density) was performed with the different untreated samples, which could reduce the variation in future experiments. When compared to the counts per milliliter provided by IFC, we see that IFC consistently measures lower concentrations. This difference is attributed to a lack of calibration in the prototype impedance flow cytometer and has been observed before [21]. However, the standard deviation is significantly lower using IFC compared to the plate counts. 

No growth was detected in the inactivated samples on the agar plates (inactivation efficiency of 100%). However, with IFC it was still possible to detect events after inactivation for all three inactivation methods. This suggests that some of the inactivated cells retain enough cell integrity after inactivation to be detected by the impedance system. In general, the lower concentration in the treated samples can be attributed to complete cell disintegration during the inactivation. Such a process is more likely to happen in *E. coli* when heat is applied, especially for a prolonged time, compared to cells exposed to ethanol [36], which corresponds well with the reduction in cell counts that we see for the three methods.

### 3.2. Characterization by Impedance

**Figure 3:**
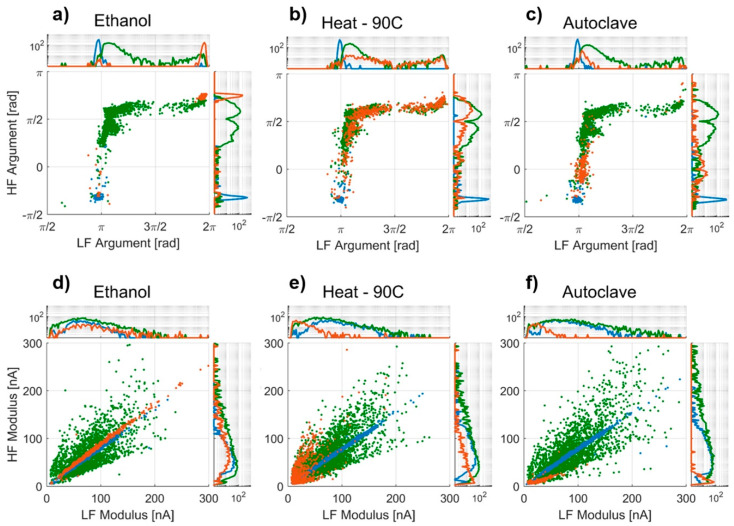
Low-frequency argument vs. high-frequency argument from the impedance measurements from the (**a**) ethanol, (**b**) heat and (**c**) autoclave experiments. Low-frequency modulus vs. high-frequency modulus from the impedance measurements from the (**d**) ethanol, (**e**) heat and (**f**) autoclave experiments. Each dot represents an event (bacteria or bead passing between the detection electrodes) with: 1.5-µm beads colored blue (●), untreated *E. coli* colored green (●), and treated *E. coli* colored orange (●). A log-scaled population density is plotted on the edges of each plot.

The measured argument and modulus of detected bacteria from each of the three inactivation experiments are shown in Figure 3, together with the measured response of a reference sample of polystyrene beads. It is evident that the beads and untreated bacteria populations, individually, appear in the same position for each of the three experiments, demonstrating that the observed changes in impedance response of the treated cells is related to the inactivation process and not the general sample handling. 

Figure 3 shows the differentiation of 1.5 µm beads, untreated *E. coli* and treated *E. coli* based on the low- and high-frequency arguments. For the ethanol and autoclave inactivation experiment we observed a significant shift between untreated and treated bacteria, except in different directions. However, no such shift was observed in the heat inactivation experiment.

The measured argument from the ethanol inactivation experiment (Figure 3a), allows us to distinguish a population for each sample with only minor overlap. The untreated bacteria and the beads show a separation between sample populations experiencing the high frequency due to the short-circuiting of the membrane capacitance, while the responses in the low-frequency condition are more similar. The untreated population of cells shows a greater variation compared to the beads as can be expected in biological samples. Comparing the ethanol-treated population to the untreated population, we observe a shift primarily in the low-frequency argument. This corresponds well with the expected response, where disruptions of the membrane lower the effective membrane resistivity and allow the electric field to penetrate the cells even at lower frequencies.

Unexpectedly, it is not possible to differentiate bacteria treated at 90 °C for 5 min from untreated bacteria (Figure 3b) in either the low- or high-frequency argument. This suggests that the treated bacteria have similar electrical properties to untreated bacteria and still retain a high degree of membrane integrity.

The population from the autoclaved sample shows a shift towards the beads in both the high-frequency and low-frequency argument, differentiating the majority of the autoclaved bacteria from the untreated bacteria (Figure 3c). Due to the high pressure and temperature of the autoclave, it is likely that the bacteria shattered and that we observed cells with highly damaged cell walls. If the cell wall is damaged to a degree in which the majority of the cytoplasm has been replaced by electrolyte, the electrical response would be similar to what we observed.

The modulus of the differential current is different for the three treated samples. The population of ethanol-treated bacteria (Figure 3d) is narrow with low variation and a slope close to 1, aligning well with the idea of a disrupted membrane, where current can flow through equally well in both low and high frequencies. 

The heat-treated sample (Figure 3e) shows a variation in modulus similar to that of the untreated bacteria but with a smaller magnitude overall. 

Similar to the heat-treated sample, the modulus for the autoclaved population (Figure 3f) is smaller compared to the untreated sample. However, for this sample we see a relatively small high frequency (HF) modulus compared to the low frequency (LF) modulus. This could support the idea that the majority of the cytoplasm has been replaced by electrolyte. At low frequencies, the disrupted membrane lets the current flow through the cell, but the intact part of the membrane still provides some resistance to the current flow. However, at higher frequencies the capacitance of the lipid membrane is short-circuited leading to cell walls that are more “transparent” and, due to the exchange of cytoplasm, a smaller differential current.

### 3.3. Fluorescence Imaging

To check whether it is indeed a loss of membrane integrity that causes the changes in impedance response, we investigated the bacteria using fluorescent staining. 

**Figure 4:**
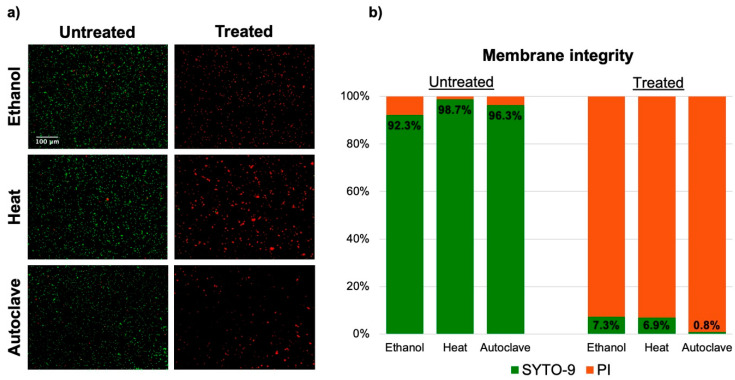
Results from the fluorescence imaging. (**a**) Images of untreated and treated bacteria from all three inactivation experiments showing green (SYTO-9) and red (PI) fluorescence. As expected, most of the untreated bacteria appear as green, while the treated bacteria appear as red. (**b**) Membrane integrity percentage for each sample (n = 3 for ethanol and heat, n = 4 for autoclave).

Images of untreated and treated fluorescent bacteria and their corresponding membrane integrity percentages are shown in Figure 4. As expected, untreated samples showed a high degree of membrane integrity across all three experiments: 92.3% for ethanol, 98.7% for heat and 94.8% for autoclave. The deviation from a 100% membrane integrity is expected to be due to a small percentage of fast-growing cells with compromised membranes [37] or bacteria that were structurally damaged during sample preparation.

The bacteria in all three treated samples exhibited low membrane integrity percentages: 7.3% for ethanol, 6.9% for heat and 1.7% for autoclave. However, these percentages are still higher than we would expect considering the plate counts (0% viability for all treatments). This could mean that a small population of bacteria keep membrane integrity but are unable to divide, or that there is an unknown intrinsic error related to the staining protocol.

These results show that all three inactivation methods used in this study—ethanol, heat and autoclave—disrupt the membrane of the bacteria. It is worth mentioning that the total number of bacteria was substantially lower in the treated samples with respect to the untreated samples. This evidence aligns with the results obtained from the plate counts for each experiment.

### 3.4. Characterization of Treated and Untreated Bacteria

**Figure 5:**
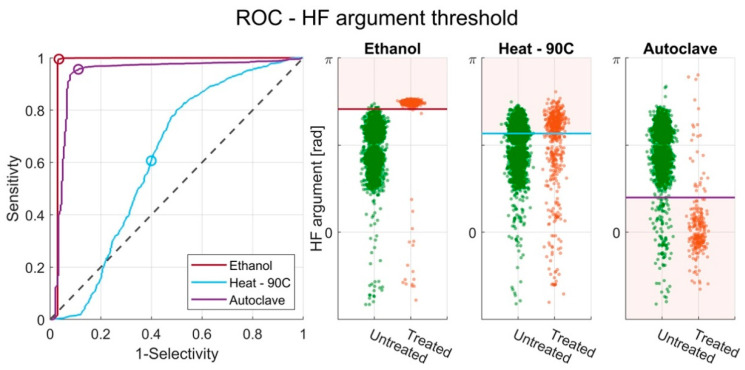
Receiver operating characteristic (ROC) curves showing the performance of the classification based on a threshold in the HF argument of treated and untreated *E. coli* for the ethanol, heat and autoclave experiments. The circles indicate thresholds of 2.22, 1.78 and 0.63 for the three experiments, respectively. The same thresholds are visualized in a categorical scatter plot showing the distribution of events in the HF argument for the untreated and treated *E. coli* for each of the three inactivation experiments.

So far, the measured current response has been investigated and correlated to the biological state of the samples. However, in order to use IFC as an alternative to plate counts (e.g., as a risk assessment tool in the food industry) it is also important to consider the effectiveness of the viability classification of the technology—i.e., investigate how well the technology can characterize viable cells as viable and how well it can characterize inactivated cells as inactivated. We therefore investigated the classification of untreated and treated bacteria for each of the three inactivation methods and determined the optimal classification threshold for each of them. We achieved this by using ROC curves as seen in Figure 5. Based on the individual thresholds for each of the inactivation methods, we found two thresholds that together return the best classification between untreated and treated bacteria across all samples. The thresholds were set in the high-frequency argument since this yielded the overall best sensitivity and selectivity. A similar analysis for the low-frequency argument can be found in the Section 2.1 of the Supplementary Materials.

#### 3.4.1. ROC with Single HF Argument Threshold

Figure 5 shows the ROC curves obtained for the HF argument from the three inactivation experiments. Optimal threshold values that classify untreated and treated *E. coli* were identified as 2.22, 1.78 and 0.63 for the ethanol, heat, and autoclavation inactivation experiments, respectively. The optimal thresholds were chosen in order to maximize sensitivity and selectivity.

The classification of untreated bacteria and bacteria treated with either ethanol or autoclavation is strong, with a sensitivity of 99.5% and 95.5%, and a selectivity of 96.7% and 88.8%, respectively. The area under the curve (AUC) values calculated for each ROC curve can be found in Table 2 and show an AUC of 0.97 and 0.93 for the ethanol experiment and autoclave experiment. However, for the heat-treated cells, the classification is quite poor with and AUC of 0.62, a sensitivity of 60.7% and a selectivity of 60.0%. This corresponds well with the observed differences in dielectric responses seen for the three experiments (Figure 3).

Included in the figure are three categorical scatter plots including the used thresholds for the HF argument. The categorical scatter plots show that the identified thresholds work well for separating untreated and treated cells in the ethanol and autoclavation experiments but not in the heat experiment. 

A summary of the AUC, sensitivity and selectivity found for each optimal threshold can be found in Table 2. 

In the Section 2.2 of the supplementary materials, three additional repetitions of the ethanol inactivation experiment are shown. The additional experiments displayed good repeatability of the results with sensitivities of 99.1%, 98.3% and 99.1%, and selectivities of 97.0%, 86.5% and 98.8%.

#### 3.4.2. ROC with Multiple HF Argument Thresholds

We have identified two thresholds (one from the ethanol inactivation experiment and one from the autoclavation experiment) that work well for classifying untreated bacteria from ethanol-treated and autoclaved bacteria, respectively. In a real-world situation, we cannot be sure how the bacteria are inactivated and therefore want to establish a general threshold that is as effective as possible for most situations. We therefore combine the two thresholds and classify every event between the thresholds as untreated bacteria and every event outside the thresholds as non-bacteria. 

**Figure 6:**
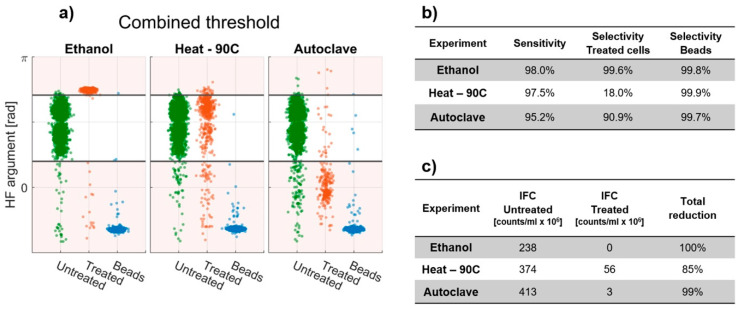
(**a**) Categorical scatter plot showing the HF argument for the untreated samples and the treated samples from the ethanol, heat and autoclave experiments. The horizontal black lines indicate the upper and lower threshold (2.22 and 0.68, respectively) used to classify viable and non-viable cells. (**b**) Sensitivity and selectivity found using the dual thresholds for each of the inactivation experiments. (**c**) Viable bacteria counts detected with Impedance Flow Cytometry (IFC) using the combined thresholds to characterize the cells for the treated and untreated samples together with the total reduction in cell count after inactivation.

The thresholds are illustrated in Figure 6a and the sensitivity and selectivity for each of the three treated samples are summarized in Figure 6b. We see that the combined thresholds are generally good at classifying untreated cells, ethanol-treated cells and autoclaved cells with sensitivities and selectivities >90%. Similarly, the selectivity of the system towards polystyrene beads was >99.7% in all three experiments. However, the combined threshold is still very poor at classifying the heat-treated cells with a selectivity of only 18%.

Even so, if the reduction in cell count from cell disruption during inactivation is combined with the improved threshold limits from the ROC analysis ($Reduction=1-\left(\frac{C_{treated}}{C_{untreated}}\right)$), the total reduction in detected viable bacteria szs found to be 100% after ethanol treatment, 85% after heat treatment and 99% after autoclavation (see Figure 6c).

Future experiments should focus on investigating the impedance response of the inactivated bacteria at additional frequencies in order to optimize the sensitivity and selectivity of the system. The full spectral sweep could be obtained by measurements using a chip with interdigitated electrodes similar to what is carried out in [16] and determining the frequencies with the highest differentiation between intact and inactivated cells. This is, of course, of particular interest when investigating the heat-inactivated samples, where improved sensitivity is crucial for the application of the technology. Special focus should be put on trying to understand why the impedance response of a heat-treated bacteria appears similar to untreated cells, even though they do not grow. It would be interesting to understand whether the number and/or size of the disruptions in the membrane play a role in this. Additionally, it would be interesting to monitor the cellular degradation of the cells over time (1–24 h) after inactivation with all three methods.

## 4. Discussion and Conclusions

In this paper we have shown how an impedance flow cytometer prototype can be used to detect and characterize *E. coli* bacteria that have been inactivated with ethanol, heat and autoclaving.

We compared the bacteria counts from IFC with those obtained from plate counts for treated and untreated bacteria and saw that the inactivation processes yielded zero CFU counts, but that the impedance flow cytometer still detected cells regardless of the viability state showcasing the increased sensitivity of the technology.

We have shown that the impedance response of bacteria changes significantly upon ethanol inactivation. This observation reinforces the belief that ethanol inactivates without necessarily dissolving the entire cell but strongly affecting the integrity of the membrane. By analyzing the impedance response of the ethanol-treated cells we were able to classify the treated cells from untreated cells with a selectivity of 99.6%.

Similarly, the impedance response of bacteria that remained after autoclaving also changed, although differently from the ethanol-inactivated cells. Here the impedance change indicates a broken cell membrane in combination with a replacement of cytoplasm with the surrounding electrolyte. By analyzing the impedance response of the autoclaved treated cells, we were able to classify the treated cells from untreated cells with a selectivity of 90.6%.

However, no significant differences were observed in the impedance response of heat-inactivated cells. This suggests that no measurable changes occurred in the cell bacteria properties, or that the system we used is not fully optimized to detect those changes. The selectivity towards treated cells was only 18.0%.

Nevertheless, investigations with fluorescent dyes showed that the cell membrane was indeed disrupted after inactivation with all three methods, proving that the structure of the cell membrane changes during inactivation. These results demonstrate that IFC can be used to detect and characterize the membrane integrity of *E. coli* bacteria, but with the current flow cytometer it is not possible for all inactivation methods. However, further investigation using different frequencies could improve this.

The need for a fast and accurate quantification of bacteria concentrations in the food industry, and other fields, is still commercially unfulfilled. This work has shown that IFC could be a promising candidate to fulfil this need, as the technology shows potential in differentiating between bacteria samples that are treated and untreated using a number of different inactivation methods.

## Figures and Tables

**Table 1:** Bacteria concentration (mL^−1^) for the treated and untreated samples calculated from drop plating and impedance measurements.

	Drop Plates (CFU/mL × 10^6^)	IFC (counts/mL × 10^6^)
**Ethanol Experiment**		
*Untreated*	520 ± 165	243 ± 47
*Treated*	0 ± 0	72 ± 8
**Heat Experiment**		
*Untreated*	590 ± 110	384 ± 12
*Treated*	0 ± 0	68 ± 3
**Autoclave Experiment**		
*Untreated*	860 ± 152	434 ± 23
*Treated*	0 ± 0	36 ± 10

**Table 2:** AUC (Area under the curve), sensitivity and selectivity found using the optimal thresholds for each of the inactivation experiments. AUC is a quality measure of the classification in general, with 1 indicating perfect classification and 0.5 indicating random classification (poor quality). The sensitivity indicates the methods ability to identify *E. coli* in the untreated sample as viable. The selectivity indicates the methods ability to identify *E. coli* in the treated samples as not viable.

	AUC	Threshold	Sensitivity	Selectivity
**Ethanol**	0.97	2.22	99.5%	96.7%
**Heat**	0.62	1.78	60.7%	60.0%
**Autoclave**	0.93	0.63	95.6%	88.8%

## Supplementary Materials

The following are available online at https://www.mdpi.com/1424-8220/20/21/6339/s1, Figure S1: (a) Schematic drawing of the impedance flow cytometer setup. A peristaltic pump continuously pumps liquid from a sample tube through the detection flow cell. The multifrequency excitation signal is pre-amplified before entering the flow cell. The measured current is differentially amplified in a trans-impedance amplifier (TIA) and the complex amplitudes from each frequency are isolated using a digital lock-in amplifier. All components are integrated in the impedance flow cytometer. A PC is used to visualize and download the recorded data. (b) Image of the impedance flow cytometer with a connected laptop. Insert shows the flow cell with the microfluidic chip mounted in a plastic casing, Figure S2: Illustration showing the bacteria preparation. A single colony from an agar plate is transferred to 40-mL tryptic soy broth (TSB) and incubated overnight in a shaking incubator at 37 °C and 200 RPM. To prepare the experimental culture, 40 µL is transferred to a fresh vial of 40-ml TSB and further incubated for ~4 h, Figure S3: Illustration showing the sample preparation for the inactivation experiment with ethanol, Figure S4: Illustration showing the sample preparation for the inactivation experiment with heat or autoclave, Figure S5: ROC curves showing the performance of the classification based on a threshold in the LF argument of treated and untreated *E. coli* for the ethanol, heat and autoclave experiments. The circles indicate thresholds of 5.98, 3.65 and 3.32 for the three experiments, respectively. The same thresholds are visualized in a categorical scatter plot showing the distribution of events in the LF argument for the untreated and treated *E. coli* for each of the three inactivation experiments, Figure S6: ROC curves showing the performance of the classification based on a threshold in the HF argument for three repetitions of the ethanol inactivation experiment. The circles mark a threshold of 2.22 corresponding to the optimal threshold for classification of untreated and ethanol-treated *E. coli*. The same threshold is also visualized in three categorical scatter plots showing the distribution of events in the HF argument for the three repetitions of the ethanol inactivation experiment, Table S1: AUC (Area under curve), Sensitivity (TPR) and Selectivity (1-FPR) found using the optimal thresholds for each of the inactivation experiments. AUC is a quality measure of the classification in general with 1 indicating perfect classification and 0.5 indicating random classification (poor quality). The sensitivity indicates the methods ability to identify *E. coli* in the untreated sample as viable. The selectivity indicates the methods ability to identify *E. coli* in the treated samples as not viable, Table S2: Area under curve (AUC), sensitivity (TPR) and selectivity (1-FPR) found using the optimal threshold for classification of untreated and ethanol-treated *E. coli* for each of the repetitions of the ethanol inactivation experiments.
