# Supplementary material for: Investigating the Use of Impedance Flow Cytometry for Classifying the Viability State of E. coli
Source: Sensors (Basel). 2020 Nov 6;20(21):6339. doi: 10.3390/s20216339 (PMC7664255; doi:10.3390/s20216339)
Supplement: Supplementary file 1 [file sensors-20-06339-s001.pdf]

sensors

Supplementary Materials

Investigating the Use of Impedance Flow Cytometry for Classifying the Viability State of *E. coli*

Christian Vinther Bertelsen [1,2,*], Julio César Franco [1], Gustav Erik Skands [2] Maria Dimaki [1] and Winnie Edith Svendsen [1]

1 DTU Bioengineering, Technical University of Denmark, Søltofts Plads 221, Kgs. Lyngby, 2800, Denmark; jucesarfranco@gmail.com (J.C.F.); madi@dtu.dk (M.D.); wisv@dtu.dk (W.E.S.)
2 SBT Instruments A/S, Symfonivej 37, Herlev 2730, Denmark; ges@sbtinstruments.com
* Correspondence: cvbe@dtu.dk

Received: 7 October 2020; Accepted: 3 November 2020; Published: date

1. Supplementary Methods

1.1. IFC Setup

A schematic overview and a photo of the experimental setup including the impedance flow cytometer (prototype, SBT Instruments, Denmark) can be seen in Figure S1. A peristaltic pump inside the flow cytometer continuously recirculates the liquid from the sample vial through the detection flow cell and back into the sample vial. Prior to entering the flow cell, the sample passes through a mesh filter that removes larger particles from the flow. The flow cell is connected to a digital lock-in amplifier via an analog trans-impedance amplifier (TIA). The stream of data is monitored in real-time on a connected computer. It is recorded on the device from where it can be downloaded after each measurement and processed for further data analysis.

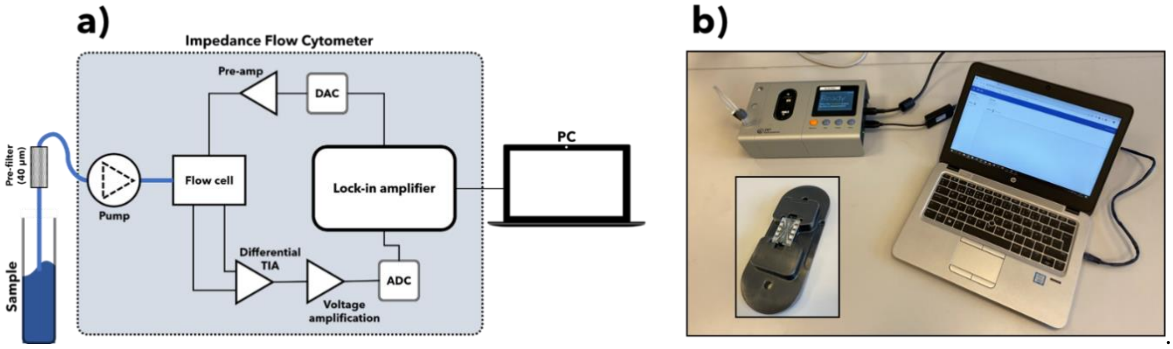

Figure S1. (**a**) Schematic drawing of the impedance flow cytometer setup. A peristaltic pump continuously pumps liquid from a sample tube through the detection flow cell. The multifrequency excitation signal is pre-amplified before entering the flow cell. The measured current is differentially amplified in a trans-impedance amplifier (TIA) and the complex amplitudes from each frequency are isolated using a digital lock-in amplifier. All components are integrated in the impedance flow cytometer. A PC is used to visualize and download the recorded data. (**b**) Image of the impedance flow cytometer with a connected laptop. Insert shows the flow cell with the microfluidic chip mounted in a plastic casing.

The detection flow cell is also provided by SBT Instruments and consists of a microfluidic chip mounted in a plastic casing with screw holes for securely mounting the flow cell in the impedance flow cytometer. The microfluidic chip connects electrically to the rest of flow cytometer through 3 spring pins (signal in and 2x signal out). The microfluidic chip is made from glass and has

microfluidic channels defined in a photosensitive polymer with platinum microelectrodes deposited on the top and bottom of the channel in a front-facing design.

The dimensions of the microfluidic channel where the detection happens is 10 × 10 µm (width x height) and the microelectrodes are 10 µm long in the flow direction of the channel and are placed 16 µm from each other (edge to edge) on the top and bottom of the channel.

Event Detection and Calculation of Concentrations

The events in the data stream were detected using a custom software program from SBT Instruments. Four peak heights (real and imaginary parts for the low and the high frequency) for each event were identified by the software and exported to a CSV file. The modulus and argument were calculated for each event using MATLAB and plotted on two scatter plots: low-frequency modulus vs. high-frequency modulus and low-frequency argument vs. high-frequency argument. The log-scaled population density was plotted on the edges of each plot in order to illustrate the density of the populations.

The modulus and argument were calculated from the real (RE) and imaginary (IM) peak values using:

$$\mathrm{abs}(\mathrm{RE}+\mathrm{i}\times\mathrm{IM}) = \sqrt{\mathrm{RE}^2+\mathrm{IM}^2} \text{ and } \arg(\mathrm{RE}+\mathrm{i}\times\mathrm{IM}) = \mathrm{atan2}(\mathrm{RE},\mathrm{IM})$$

with

$$\mathrm{atan2}(\mathrm{IM},\mathrm{RE}) = \begin{cases} \arctan\left(\frac{\mathrm{IM}}{\mathrm{RE}}\right), & \text{if RE} > 0 \\ \arctan\left(\frac{\mathrm{IM}}{\mathrm{RE}}\right) + \pi, & \text{if RE} < 0 \text{ and IM } \geq 0 \\ \arctan\left(\frac{\mathrm{IM}}{\mathrm{RE}}\right) - \pi, & \text{if RE} < 0 \text{ and IM} < 0 \\ +\frac{\pi}{2}, & \text{if RE} = 0 \text{ and IM} > 0 \\ -\frac{\pi}{2}, & \text{if RE} = 0 \text{ and IM} < 0 \\ \text{undefined}, & \text{if RE} = 0 \text{ and IM} = 0 \end{cases}$$

1.2. Experimental Procedure

A graphical illustration of the bacteria preparation and inactivation experiments can be seen in Figure S2, Figure S3 and Figure S4, respectively.

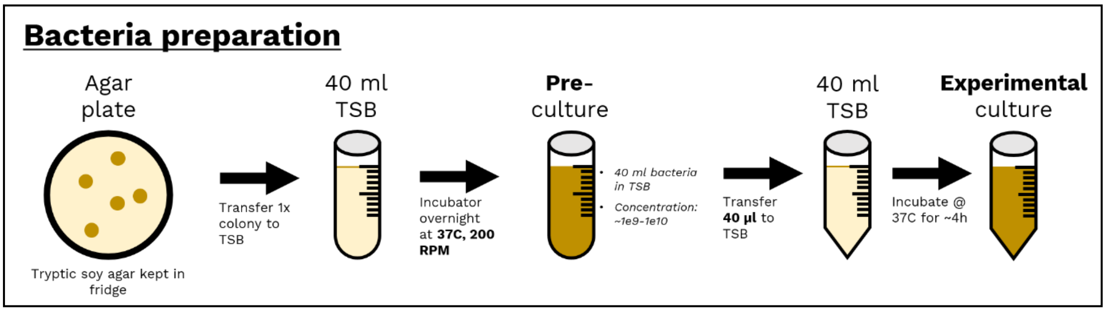

Figure S2. Illustration showing the bacteria preparation. A single colony from an agar plate is transferred to 40-ml tryptic soy broth (TSB) and incubated overnight in a shaking incubator at 37ºC and 200 RPM. To prepare the experimental culture, 40 µl is transferred to a fresh vial of 40-ml TSB and further incubated for ~4 hours.

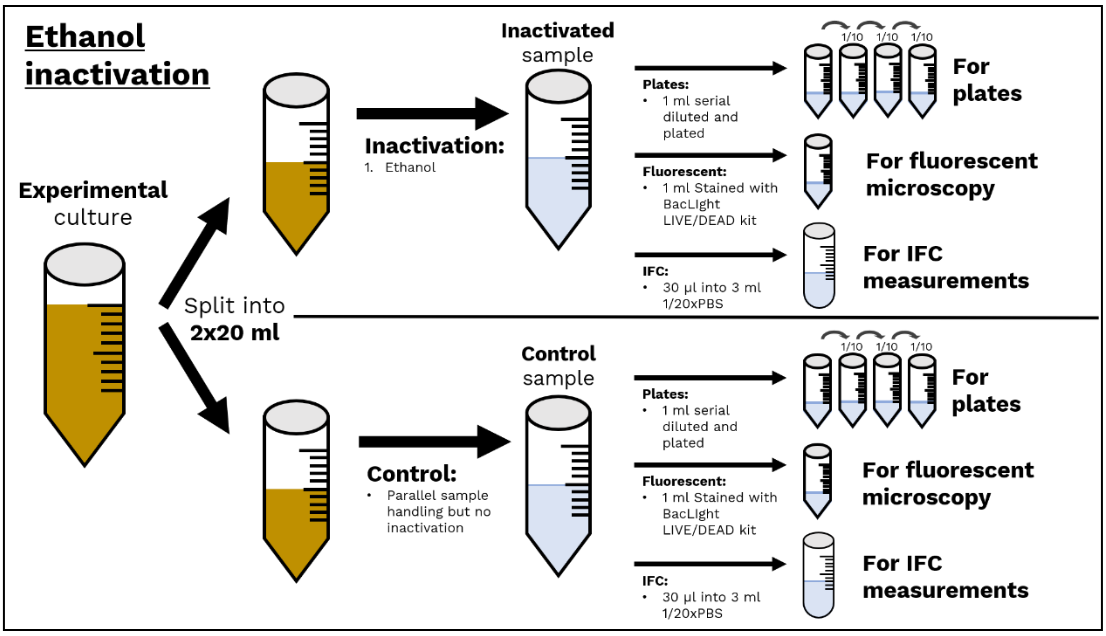

Figure S3. Illustration showing the sample preparation for the inactivation experiment with ethanol.

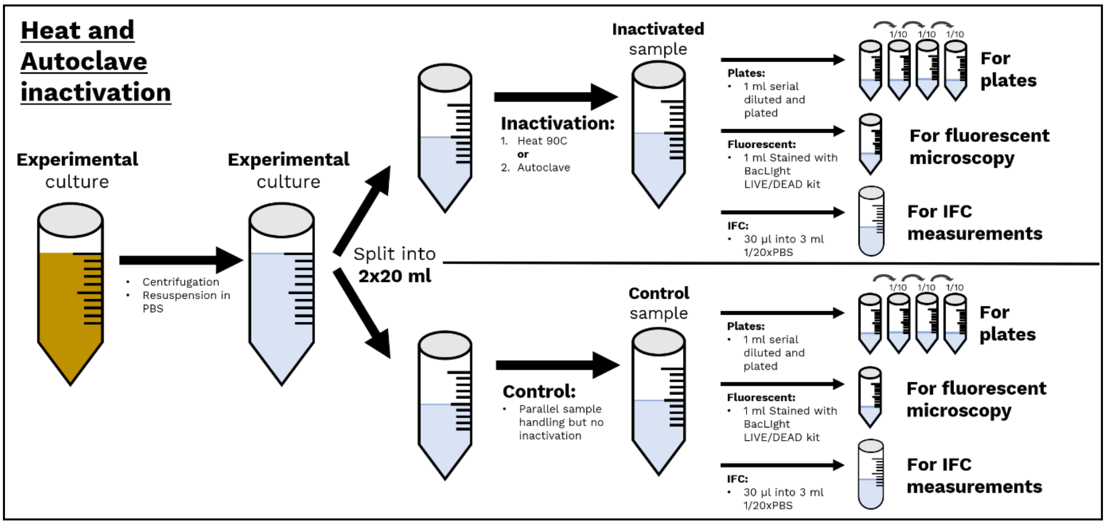

Figure S4. Illustration showing the sample preparation for the inactivation experiment with heat or autoclave.

1.3. Processing of Fluorescent Images

The image processing consisted of an enhancement of the brightness and the contrast using Fiji ImageJ [1]. Bacteria counts were performed using the threshold and the analyze particles functions. Bacteria counts were performed in both the green fluorescent and the red fluorescent images and both counts were used to calculate viability rates. The green and red superimposed fluorescence images were obtained by merging both channel colors using the merge channels function. For the scale setting, a Neubauer-improved chamber (Marienfeld, Germany) was used.

2. Supplementary Results

2.1. ROC—Low Frequency Argument Threshold

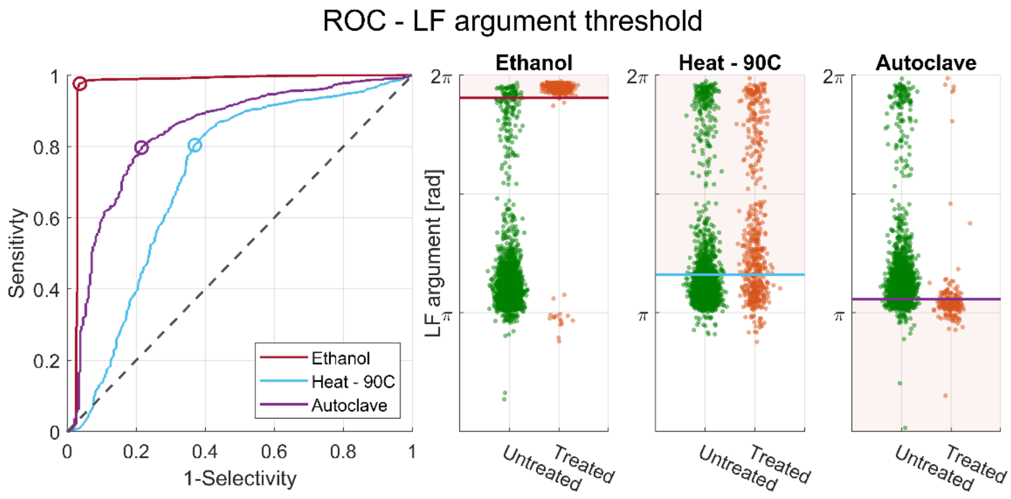

Figure S5. ROC curves showing the performance of the classification based on a threshold in the LF argument of treated and untreated E.coli for the ethanol, heat and autoclave experiments. The circles indicate thresholds of 5.98, 3.65 and 3.32 for the three experiments, respectively. The same thresholds are visualized in a categorical scatter plot showing the distribution of events in the LF argument for the untreated and treated E. coli for each of the three inactivation experiments.

Table S1. AUC (Area under curve), Sensitivity (TPR) and Selectivity (1-FPR) found using the optimal thresholds for each of the inactivation experiments. AUC is a quality measure of the classification in general with 1 indicating perfect classification and 0.5 indicating random classification (poor quality). The sensitivity indicates the methods ability to identify E. coli in the untreated sample as viable. The selectivity indicates the methods ability to identify E. coli in the treated samples as not viable.

	AUC	Threshold	Sensitivity	Selectivity
Ethanol	0.97	5.98	97.6%	96.3%
Heat—90℃	0.72	3.65	80.4%	63.3%
Autoclave	0.84	3.32	79.7%	78.5%

2.2. Repetitions of Measurements with Ethanol Inactivation

The ROC curves show that IFC can distinguish between viable E. coli and E. coli that has been inactivated with ethanol very well using a threshold value of 2.22 in the high-frequency argument. However, if IFC is going to be a useful technology for bacteria enumeration (for example, in food production), the repeatability of the classification using the same threshold needs to be high. In order to investigate how consistently IFC separates viable cells from ethanol-inactivated cells we repeated the experiment with ethanol inactivation an additional three times.

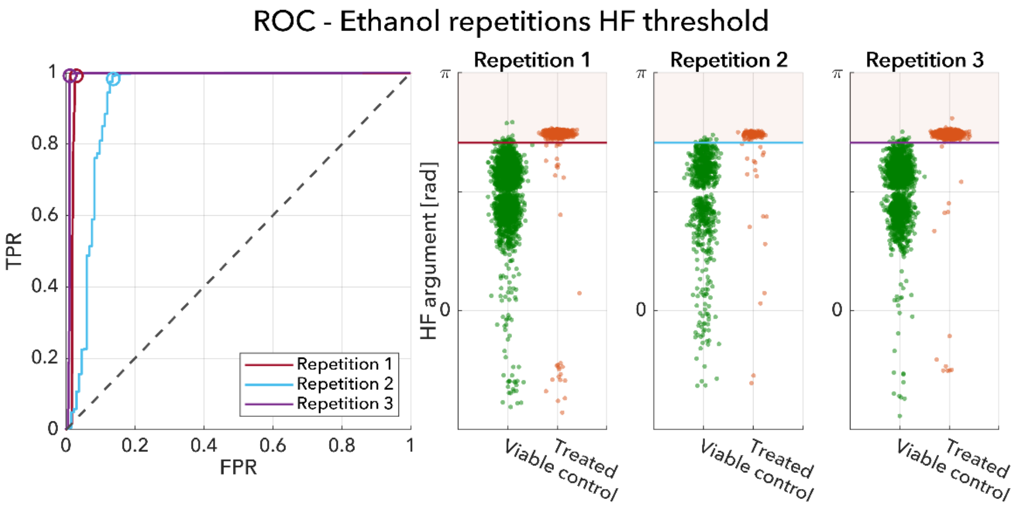

Figure S6. ROC curves showing the performance of the classification based on a threshold in the HF argument for three repetitions of the ethanol inactivation experiment. The circles mark a threshold of 2.22 corresponding to the optimal threshold for classification of untreated and ethanol-treated E. coli. The same threshold is also visualized in three categorical scatter plots showing the distribution of events in the HF argument for the three repetitions of the ethanol inactivation experiment.

Figure 6 shows the ROC-plots of three repetitions of the experiment with untreated and ethanol-treated *E. coli*. The optimal threshold of 2.22 found in the first experiment is marked with a line for each repetition. The AUC, sensitivity and selectivity obtained using a threshold of 2.22 in the high-frequency argument is shown in Table 2. We observed that the IFC system had good repeatability across experiments, although it is not perfect with the selectivity dropping to 86.5% in the second repetition compared to 97.0% and 98.8% in the other two repetitions. However, in general, the sensitivity and selectivity were very acceptable in all three repetitions.

Table S2. Area under curve (AUC), sensitivity (TPR) and selectivity (1-FPR) found using the optimal threshold for classification of untreated and ethanol-treated E. coli for each of the repetitions of the ethanol inactivation experiments.

	**AUC**	**Threshold**	**Sensitivity**	**Selectivity**
Ethanol repetition 1	0.98	2.22	99.1%	97.0%
Ethanol repetition 2	0.93	2.22	98.3%	86.5%
Ethanol repetition 3	0.99	2.22	99.1%	98.8%
